# State-of-the-Art and New Treatment Approaches for Spinal Cord Tumors

**DOI:** 10.3390/cancers16132360

**Published:** 2024-06-27

**Authors:** Chetan Kumawat, Toshiyuki Takahashi, Isao Date, Yousuke Tomita, Masato Tanaka, Shinya Arataki, Tadashi Komatsubara, Angel O. P. Flores, Dongwoo Yu, Mukul Jain

**Affiliations:** 1 Department of Orthopedic Surgery, Okayama Rosai Hospital, 1-10-25 Chikkomidorimachi, Minami Ward Okayama, Okayama 702-8055, Japan; dr.ckumawat@gmail.com (C.K.); araoyc@gmail.com (S.A.); t.komatsubara1982@gmail.com (T.K.); angeloscarpaz@gmail.com (A.O.P.F.); icarus0810@hanmail.net (D.Y.); drmukuljain92@gmail.com (M.J.); 2 Department of Orthopedic Surgery, Sir Ganga Ram Hospital, Rajinder Nagar, New Delhi 110060, India; 3 Spinal Disorder Center, Fujieda Heisei Memorial Hospital, 123-1 Mizuue Fujieda, Shizuoka 426-8662, Japan; heisei.t-taka@ny.tokai.or.jp; 4 Department of Neurosurgery, Okayama Rosai Hospital, 1-10-25 Chikkomidorimachi, Minami Ward Okayama, Okayama 702-8055, Japan; idate333@md.okayama-u.ac.jp (I.D.); tomitamiharayosu@gmail.com (Y.T.)

**Keywords:** spinal cord tumor, astrocytoma, diagnosis, current treatment, surgery

## Abstract

**Simple Summary:**

Spinal cord tumors encompass a diverse range of rare neoplasms originating from tissues in and around the spinal canal. Traditional treatment modalities like surgery, radiation therapy, and chemotherapy have been the mainstay for managing these tumors. Nowadays, advancements in gene therapy, immunotherapy, and targeted therapy are offering groundbreaking possibilities. This article outlines the available and developing options for diagnosis and such treatments.

**Abstract:**

Spinal cord tumors, though rare, present formidable challenges in clinical management due to their intricate nature. Traditional treatment modalities like surgery, radiation therapy, and chemotherapy have been the mainstay for managing these tumors. However, despite significant advancements, challenges persist, including the limitations of surgical resection and the potential side effects associated with radiation therapy. In response to these limitations, a wave of innovative approaches is reshaping the treatment landscape for spinal cord tumors. Advancements in gene therapy, immunotherapy, and targeted therapy are offering groundbreaking possibilities. Gene therapy holds the potential to modify the genes responsible for tumor growth, while immunotherapy harnesses the body’s own immune system to fight cancer cells. Targeted therapy aims to strike a specific vulnerability within the tumor cells, offering a more precise and potentially less toxic approach. Additionally, novel surgical adjuncts are being explored to improve visualization and minimize damage to surrounding healthy tissue during tumor removal. These developments pave the way for a future of personalized medicine for spinal cord tumors. By delving deeper into the molecular makeup of individual tumors, doctors can tailor treatment strategies to target specific mutations and vulnerabilities. This personalized approach offers the potential for more effective interventions with fewer side effects, ultimately leading to improved patient outcomes and a better quality of life. This evolving landscape of spinal cord tumor management signifies the crucial integration of established and innovative strategies to create a brighter future for patients battling this complex condition.

## 1. Introduction

Spinal cord tumors encompass a diverse range of rare neoplasms originating from tissues in and around the spinal canal. These tumors typically exhibit a benign onset with a gradual progression of signs and symptoms. They are characterized by histological heterogeneity, indicating potential origination from various precursor cells [1,2]. Constituting a mere 2–4% of all primary tumors within the central nervous system (CNS), these tumors present a significant clinical challenge [3,4,5]. Despite their lower incidence compared with their intracranial counterparts, spinal cord tumors share histopathological similarities with primary intracranial neoplasms, emphasizing the need for a nuanced approach to their management [4]. By convention, spinal cord tumors are commonly classified by anatomic sublocation as intradural intramedullary, intradural extramedullary, or extradural [1] (Table 1). Extradural spinal tumors are the most prevalent tumors of the spine, constituting 50% [6]. They are further categorized into primary and secondary tumors. Primary extradural tumors, such as hemangiomas and enostoses, are exceptionally rare, often incidental findings, and frequently asymptomatic, requiring no treatment [7]. In contrast, secondary tumors constitute 97% of all vertebral spinal tumors due to the spine’s rich vascularity and proximity to lymphatic drainage [7].

Intradural extramedullary spinal cord tumors (EMSCTs) develop in the subdural space, outside of the spinal cord, accounting for 40% [6]. These tumors are from leptomeninges or nerve roots [8]. Among EMSCTs, schwannomas are the most common, constituting 29% [9]. Patients with EMSCTs often have lower back pain, especially worsened in the supine position [9]. Intramedullary spinal cord tumors (IMSCTs) constitute 5–10% of tumors and gliomas constitute up to 90%, including ependymomas (two-thirds) and astrocytomas (one-third) [10,11,12]. 

The classification of CNS tumors has traditionally relied on histological analysis, supplemented by ancillary tests such as immunohistochemistry and ultrastructural studies. However, in recent years, molecular biomarkers have emerged as crucial tools in enhancing diagnostic accuracy and refining classification criteria. The latest edition of the WHO Classification of Tumors of the Central Nervous System (CNS) integrates a multitude of molecular alterations that provide valuable diagnostic and prognostic insights. These molecular changes not only complement histological findings but also offer defining information, thereby contributing to a more precise and comprehensive classification of CNS neoplasms [13] (Table 1 and Table 2).

**Table 1:** WHO classification (grading) [13].

Grade 1	These are the least malignant tumors and are usually associated with long-term survival. They grow slowly and have an almost normal appearance when viewed through a microscope. Surgery alone may be an effective treatment for this grade tumor.
Grade 2	These tumors are slow-growing and look slightly abnormal under a microscope. Some can spread into nearby normal tissue and recur, sometimes as a higher-grade tumor.
Grade 3	These tumors are, by definition, malignant, although there is not always a big difference between grade 2 and grade 3 tumors. The cells of a grade 3 tumor are actively reproducing abnormal cells, which grow into nearby normal brain tissue. These tumors tend to recur, often as a grade 4 tumors.
Grade 4	These are the most malignant tumors. They reproduce rapidly, can have a bizarre appearance when viewed under a microscope, and easily grow into nearby normal brain tissue. These tumors form new blood vessels so they can maintain their rapid growth. They also have areas of dead cells in their centers.

**Table 2:** WHO classification (origin) [13].

Neuroepithelial tissue	
Paraspinal nerves	
Meninges	Meningothelial cells; mesenchymal; primary melanocytic lesions; other neoplasms
Lymphoma and hematopoietic neoplasms	
Germ cell tumors	
Metastatic tumors	

Navigating the intricate landscape of spinal cord tumors, characterized by their rarity and complexity, demands a comprehensive understanding of both established state-of-the-art treatment modalities and emerging innovative approaches. Various surgical techniques can be employed for tumor excision, depending on factors such as the tumor type, surgical goal, and the patient’s overall health status [14]. Traditional treatment modalities, including surgery, radiation therapy, and targeted drug therapies, have formed the backbone of spinal cord tumor management [3,5,15]. Surgical resection, guided by advances in imaging and neurosurgical techniques, remains a primary intervention, aiming for maximal tumor removal while preserving neurological function [3,15]. Radiation therapy, encompassing conventional external beam radiation and modern techniques like stereotactic radiosurgery, plays a crucial role in adjuvant and palliative settings [3]. Additionally, targeted drug therapies, leveraging molecular insights into tumor biology, offer promising avenues for personalized treatment approaches [15]. However, despite the progress achieved with traditional treatment modalities, challenges persist in achieving optimal outcomes for patients with spinal cord tumors. These challenges include limitations in surgical resection due to tumor location or size and the risk of damaging surrounding healthy tissues with radiation therapy [3].

Amidst these challenges, the field of spinal cord tumor research is witnessing a surge of innovative approaches aimed at addressing existing limitations and enhancing therapeutic efficacy. Immunotherapy, harnessing the body’s immune system to recognize and attack tumor cells, offers a paradigm shift toward personalized and potentially curative treatment strategies [16,17]. The cytotoxic capabilities of the immune system and the precision afforded by molecular targeting make immunotherapy promising [18].To enhance the precision of procedures and improve postoperative outcomes, emerging digital technologies are increasingly being integrated into tumor resections as complementary tools for visualizing the surgical field [19]. In this review, we will explore how the fusion of established state-of-the-art treatment methods with emerging innovative approaches opens up vast promise and potential in the management of spinal cord tumors, enriching our understanding of this complex field. By seamlessly integrating these strategies, the field aspires to surpass current limitations, leading the path toward a future where patients with spinal cord tumors can access refined treatment protocols and enjoy an enhanced quality of life.

## 2. Diagnosis

### 2.1. MRI and CT

Diagnosing IMSCTs and EMSCTs is complex due to their diverse behaviors, which can range from benign to malignant [20]. These tumors often elude early detection and only present neurological symptoms after substantial infiltration of the spinal canal, resulting in considerable morbidity and mortality [21]. Consequently, reliable and timely diagnostic techniques are critical for effective treatment. Magnetic resonance imaging (MRI) is the primary diagnostic tool for identifying spinal tumors, providing detailed information about their size, location, and position along the axis [22].

Table 3, in the context of spinal cord tumors, serves as a guide for clinicians to differentiate between various types of spinal cord tumors based on specific imaging and clinical characteristics.

Tumor location: The location of the tumor within the spinal cord or surrounding structures can provide valuable information about its possible origin and nature. For example, tumors located within the spinal cord parenchyma may indicate intramedullary tumors, while those located outside the cord may suggest extramedullary tumors. I IMSCTs include ependymomas and astrocytomas. EMSCTs include meningiomas and schwannomas [1,6].

MRI intensity and CT density: The intensity or density of the tumor in magnetic resonance imaging (MRI) or computed tomography (CT) scans can offer insights into its composition and characteristics. Different types of tumors may exhibit distinct intensity or density patterns, aiding in their differentiation. Low-grade gliomas (e.g., astrocytomas) may appear hypointense in T1-weighted MRI and hyperintense in T2-weighted MRI. Meningiomas often demonstrate the isointensity in T1-weighted MRI and hyperintensity in T2-weighted MRI [23]. Conventional MRI only provides anatomical information. However, MR diffusion tensor imaging (DTI) and MR perfusion-weighted imaging (PWI) may detect microstructural diffusion and hemodynamic changes in spinal cord tumors. These techniques are useful for improving differential diagnoses between spinal cord tumors and tumor mimics especially for demyelinating disease, tumor grading, and providing assistance in surgical navigation [24]. The most common MRI perfusion techniques utilized are dynamic susceptibility contrast (DSC), arterial spin labeling (ASL), and dynamic contrast-enhanced (DCE) perfusion. Susceptibility-weighted imaging (SWI) is a high-spatial-resolution, three-dimensional (3D), fully velocity-compensated, gradient echo MRI technique that accentuates the magnetic properties (susceptibility effects) of various substances such as blood products, calcification, and iron [25]. Magnetic resonance spectroscopy (MRS) can aid in the evaluation of pediatric brain tumors by providing metabolic information complementary to neuroanatomical imaging [26].

Enhancement pattern: The enhancement pattern observed in contrast-enhanced imaging studies can help distinguish between various types of spinal cord tumors. For instance, certain tumors may show homogeneous enhancement, while others may exhibit heterogeneous enhancement patterns. Ependymomas may exhibit heterogeneous enhancement with contrast due to the presence of cystic components. Schwannomas typically show intense, homogeneous enhancement following contrast administration [27].

Bone erosion: The presence of bone erosion detected in imaging studies, such as CT scans, may suggest an invasive tumor that has extended into the surrounding bone tissue. This finding can help narrow down the differential diagnosis and guide treatment planning. Chordomas are known to cause bone erosion and destruction of adjacent vertebral bodies due to their locally aggressive nature. Metastatic spinal tumors, such as from lung or breast cancer, may also lead to bone erosion as they invade the vertebral column [28,29].

Accompanied findings: Additional imaging findings, such as peritumoral cysts, edema, flow voids, or calcifications, can provide further clues about the nature and characteristics of a tumor. These accompanying features may vary depending on the tumor type and location. Peritumoral cysts: These are seen in ependymomas and hemangioblastomas [27,30]. Flow voids: These are characteristic of vascular tumors like spinal hemangioblastomas [30]. Edema: These are commonly observed around high-grade gliomas, such as glioblastomas [31]. Calcifications: These are seen in oligodendrogliomas and meningiomas [32].

Other studies: In some cases, supplementary studies such as angiography, positron emission tomography (PET), or cerebrospinal fluid (CSF) analysis may be necessary to confirm the diagnosis or rule out other possibilities. These additional investigations can contribute to a more comprehensive evaluation and management plan for spinal cord tumors. Angiography: This is helpful in delineating vascular malformations or tumors with a prominent vascular supply, such as hemangioblastomas. PET scan: This can aid in detecting metabolic activity and differentiating between benign and malignant tumors. CSF study: This may be indicated to evaluate for leptomeningeal involvement or detect tumor markers in cerebrospinal fluid, particularly in cases of suspected metastatic disease or primary CNS lymphoma [33].

Among EMSCTs, meningiomas are typically iso- or hypointense in T1-weighted MRI scans and mildly hyperintense in T2-weighted scans, with most showing a “dural tail” sign after gadolinium contrast enhancement [23] Additionally, CT myelography is used to detect calcifications within tumors or when MRI is not suitable. In specific scenarios, spinal angiography is utilized preoperatively to facilitate the embolization of the arteries supplying the tumor, thereby reducing intraoperative bleeding and tumor size.

**Figure 1:**
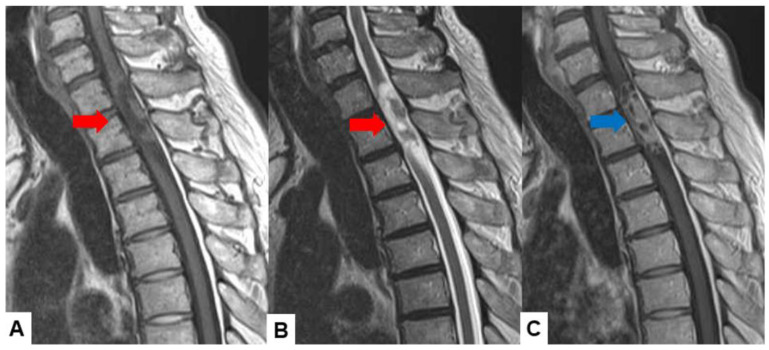
Fifty-four M, spinal schwannoma. (**A**) T1-weighted midsagittal image, (**B**) T2-weighted midsagittal image, and (**C**) enhanced T1-weighted midsagittal image. Red arrows indicate tumor and blue arrow shows tumor enhancement. The tumor is mixed-intensity because of tumor necrosis.

**Figure 2:**
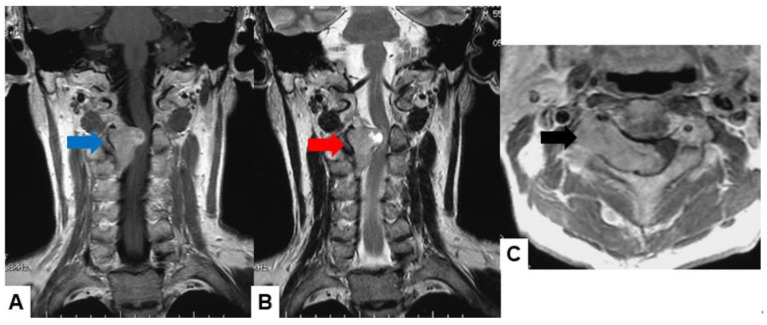
Fifty-five M, spinal schwannoma. (**A**) Enhanced T1-weighted midsagittal image, (**B**) T2-weighted midsagittal image, and (**C**) enhanced T1-weighted axial image at C2/3. Red arrow indicates tumor and blue arrow shows tumor enhancement. The tumor is dumbbell-shaped (black arrow).

Schwannomas usually show low intensity in T1-weighted MR imaging and high intensity in T2-weighted MR imaging (Figure 1 and Figure 2). These tumors often enlarge alongside the nerve root and become a dumbbell-type [34]. The heterogenicity of a tumor indicates cystic change. 

**Figure 3:**
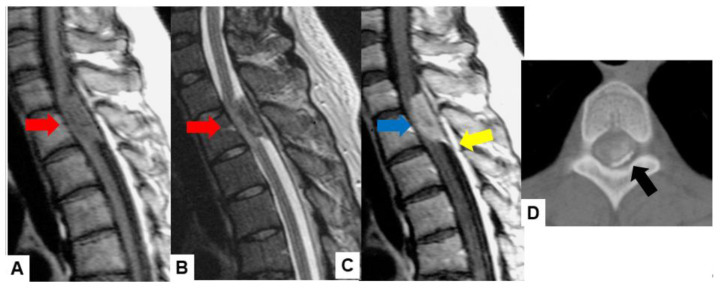
Forty-two F, spinal meningioma. (**A**) T1-weighted midsagittal image, (**B**) T2-weighted midsagittal image, and (**C**) enhanced T1-weighted midsagittal image. (**D**) CT. Red arrows indicate tumor and blue arrow shows tumor enhancement. Yellow arrow shows dural tail sign and black arrow indicates tumor ossification.

**Figure 4:**
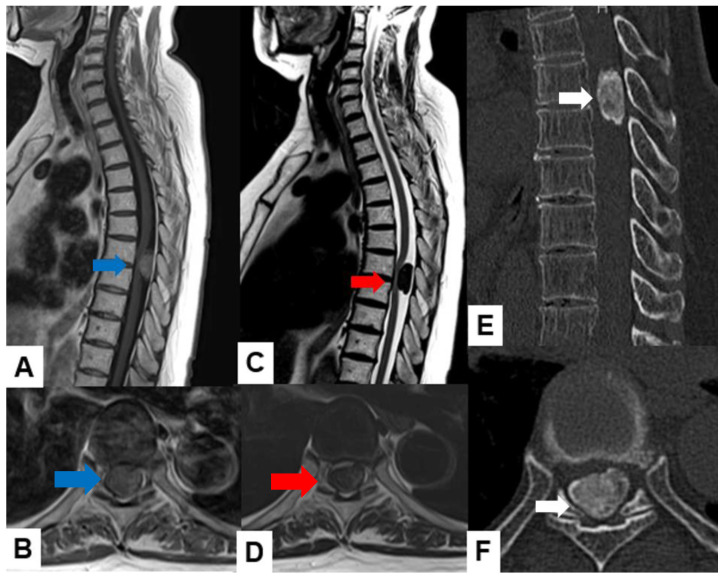
Sixty-eight F, spinal meningioma. (**A**) Enhanced T1-weighted midsagittal image, (**B**) enhanced T1-weighted axial image at T7/8, (**C**) T2-weighted midsagittal image, (**D**) T2-weighted axial image at T7/8, (**E**) midsagittal reconstruction CT, and (**F**) axial CT at T7/8. Red arrows indicate tumor, and blue arrows show tumor enhancement. The tumor is calcified (white arrows).

Meningiomas usually indicate isointensity in both T1- and T2-weighted MR imaging and are enhanced homogeneously (Figure 3) [35]. Meningiomas are sometimes calcified and are recognized in CT (Figure 4). The key points of a differential diagnosis of meningioma and schwannoma are in Table 4.

The characterization of IMSCTs involves distinguishing the tumor from surrounding edema or cavities, which provides crucial information about its position, size, and growth dynamics (Table 5 and Table 6). Addressing the challenges of manual segmentation, Lemay et al. developed an automated technique using a cascaded architecture based on U-Net models [36]. This approach simplifies the segmentation process into two phases of precise localization and labeling, improving the efficiency and accuracy of tumor identification. 

Furthermore, the integration of emerging technologies like artificial intelligence (AI) and machine learning is poised to enhance diagnostic precision and accelerate treatment planning for IMSCTs [37]. The adoption of these technologies in clinical settings could transform diagnostic procedures, promoting earlier interventions and better patient outcomes for those with spinal cord tumors.

**Table 5:** Spinal intramedullary tumors in adults [2].

Tumor	Incidence
Ependymoma	50–60%
-Myxopapillary	20–30%
Astrocytoma	15–30%
-Pilocytic	10–45%
-High-grade	10–33%
Hemangioblastoma	3–11%
Cavernous angioma	4–5%
Schwannoma	1%
Metastasis	1%

**Table 6:** Spinal intramedullary tumors in children [38].

Tumor	Incidence
Astrocytoma	41%
-Pilocytic	6%
-High-grade	26%
Ganglioglioma	27%
Ependymoma	12%
-Myxopapillary	35%
Hemangioblastoma	2%

**Figure 5:**
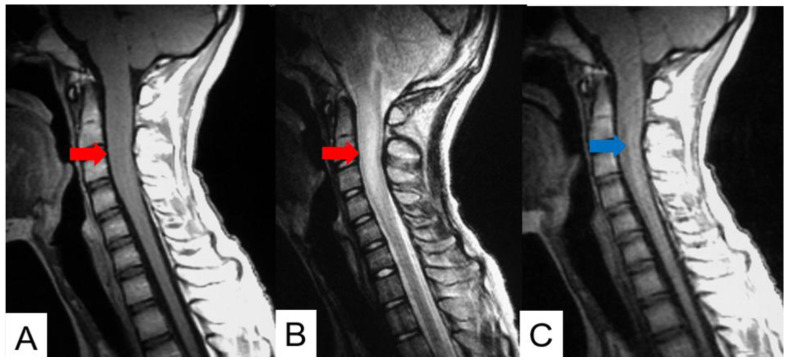
Fourteen M, spinal astrocytoma, grade 3. (**A**) T1-weighted midsagittal image, (**B**) T2-weighted midsagittal image, and (**C**) enhanced T1-weighted midsagittal image. Red arrows indicate tumor and blue arrow shows tumor enhancement.

Astrocytoma: Pilocytic astrocytomas usually indicate well-circumscribed intramedullary masses with cystic lesions. These tumors exhibit a mixed signal intensity in both T1-weighted and T2-weighted images (Figure 5). High-grade astrocytomas, such as glioblastomas, present as infiltrative growth, with ill-defined margins and heterogeneous enhancement. These tumors may indicate surrounding edema. 

**Figure 6:**
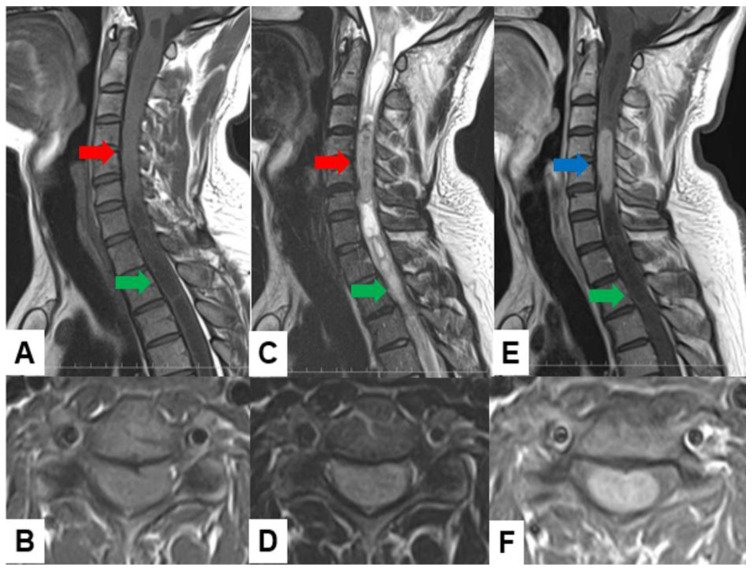
Thirty-seven M, spinal ependymoma, grade 1. (**A**) T1-weighted midsagittal image, (**B**) T1-weighted axial image at C4/5, (**C**) T2-weighted midsagittal image, (**D**) T2-weighted axial image at C4/5, (**E**) enhanced T1-weighted midsagittal image, and (**F**) enhanced T1-weighted axial image at C4/5. Red arrows indicate tumor; blue arrow shows enhancement. Green arrows indicate large syringomyelia.

Ependymoma: Myxopapillary ependymomas typically present as well-defined intradural extramedullary masses with a heterogeneous signal intensity in T1-weighted images and hyperintensity in T2-weighted images (Figure 6). These tumors often demonstrate avid contrast enhancement.

**Figure 7:**
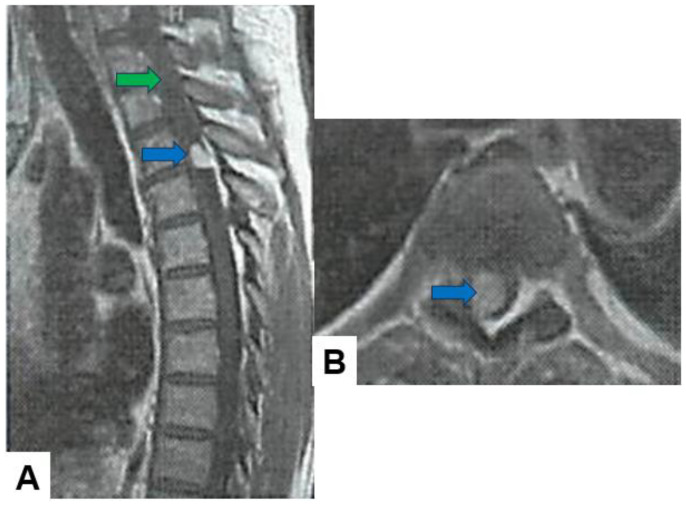
Fifty-four M, spinal hemangioblastoma, grade 1. (**A**) Enhanced T1-weighted midsagittal image; (**B**) enhanced T1-weighted axial image at T2/3. Blue arrows indicate tumor enhancement and green arrow shows syringomyelia.

Hemangioblastoma: Hemangioblastomas usually appear as well-circumscribed intramedullary lesions with marked hypointensity in T1-weighted images and hyperintensity in T2-weighted images due to the presence of cystic components and vascularity. They typically demonstrate avid contrast enhancement (Figure 7).

**Figure 8:**
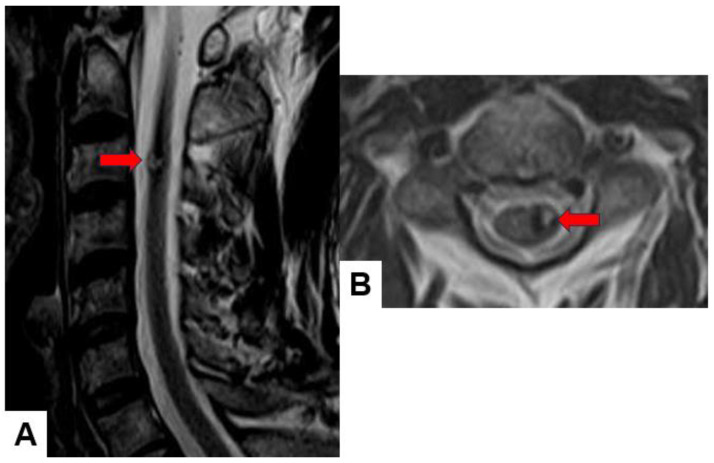
Forty-nine M, cavernous angioma. (**A**) T2-weighted midsagittal image; (**B**) T2-weighted axial image at C3. Red arrows indicate tumor.

Cavernous angioma: Cavernous angiomas present as well-circumscribed intramedullary lesions with a mixed signal intensity in both T1-weighted and T2-weighted images (Figure 8), often showing a characteristic “popcorn” appearance due to multiple blood-filled caverns. They may demonstrate variable enhancement patterns.

Metastasis: Metastatic spinal cord tumors often present as multiple intramedullary or intradural extramedullary lesions with a variable signal intensity in both T1-weighted and T2-weighted images, depending on the primary tumor’s histology. They may demonstrate avid contrast enhancement and typically show associated vertebral body metastases.

### 2.2. Molecular and Genetic Profiling

Beyond diagnostic neuroradiology, molecular genomics is increasingly employed to decode the complexities of spinal cord tumors. Research, such as the work of Jung et al., analyzing clinical and radiological data to predict H3 K27M mutations, and Pandey et al., developing techniques to differentiate between driver and passenger mutations in glioblastomas, demonstrates the potential of these approaches [39,40]. These methods help prioritize essential mutations and inform the development of targeted treatments. The genetic and molecular characterization of spinal cord tumors, especially astrocytomas and ependymomas, is challenging due to their rarity and the difficulty in obtaining sufficient samples for thorough analysis compared with brain tumors. Nonetheless, recent progress has illuminated several common and distinct genetic features among these tumor types. For example, spinal ependymomas commonly show mutations in the NF2 gene, whereas spinal meningiomas often have chromosomal irregularities, including the loss of chromosome 22 [41,42]. Additionally, schwannomas and neurofibromas are tightly linked with genetic conditions such as neurofibromatosis type 1 (NF1) and NF2, with schwannomatosis emerging as a separate syndrome associated with SMARCB1 mutations [43,44,45]. These molecular insights are guiding the development of targeted treatments and personalized care strategies. Overall, molecular and genetic analysis is proving essential for enhancing the diagnosis and treatment of spinal cord tumors.

### 2.3. The Important Role of Biopsy and Deferential Diagnosis of Other Non-Tumor Conditions

A biopsy plays one of the most important roles in the diagnosis of spinal oncology. Although modern imaging technology is well-developed, a precise histological diagnosis is essential for further treatment [45]. For a vertebral lesion, a percutaneous core needle biopsy is suitable for spinal vertebral lesions [46]. Recently, when core needle biopsies were performed under CT guidance, they were demonstrated to be more accurate and safer for patients than C-arm-guided biopsies.

Usually, a spinal cord biopsy is contraindicated because the procedure causes seriously compromised spinal cord function. Especially, in patients with known malignancy who present with intramedullary lesions, as in this situation, the diagnosis can be made with the clinical setting [47]. Also, this procedure is not recommended in the evaluation of autoimmune myelopathies associated with AQP4-IgG because the risks outweigh the benefits [48]. However, fine-needle aspiration of mass lesions in the vertebral canal can be useful and produces little damage. For the diagnosis of sarcoidosis, a biopsy of sites outside the central nervous system is preferable, but if no other sites are available and diagnostic certainty is important, sometimes a spinal cord biopsy is necessary to confirm the diagnosis of isolated spinal cord sarcoidosis [49]. 

The differential diagnosis of spinal oncology is very important in congenital or other conditions. A neurenteric cyst is a rare lesion of the spinal axis composed of heterotopic endodermal tissue, and its incidence is 0.7–1.3% of all spinal cord tumors [50]. Wilkins et al. classified neurenteric cysts based on three histopathological presentations [51] (Table 7). Patients with symptomatic neurenteric cysts typically present in the second and third decades of life with myelopathy and/or radicular symptoms. The surgical results are relatively good with minimal morbidity [52].

The ventriculus terminalis is an embryological remnant consisting of the ependymal-lined space of the conus medullaris. This anomaly can become symptomatic after cystic dilation. de Moura and Ganau established a clinical classification system based on the available literature as the cystic lesion of the ventriculus terminalis classification (CLVT): type Ia (stable nonspecific symptoms without a clear relation to the ventriculus terminalis), type Ib (nonspecific but progressing symptoms), type II (focal neurological deficits), and type III (sphincter disturbances) [53,54]. Type Ia is best treated conservatively, and type Ib may benefit from surgical evacuation. In patients with type II (focal neurological deficits) and III (sphincter disturbances) symptoms, surgical treatment sustained improvement even at the late follow-up [54].

Spinal cord infection is a rare disease with different etiological causes. In an acute presentation, a differential diagnosis is relatively easy because of the symptoms of infection (fever and severe pain) and increased white blood count and CRP. However, chronic cases might mimic features of intramedullary tumors and show neurological symptoms [55]. Spinal cord ischemia is also a rare disease and constitutes one of the acute spinal emergencies. The spinal cord is usually enlarged, and MRI indicates hyperintensity in T2-weighted images and DWI [56]. This may occur at any location in the cord but has a propensity for the upper thoracic or thoracolumbar regions [57].

Vascular malformations may also mimic a spinal cord tumor. Spinal AVMs are a heterogeneous group of abnormally developed spinal blood vessels associated with an increased risk for hemorrhage and morbidity [58]. Due to the shunting of arteriole blood to the venous system without capillary access and resistance, over 70% of arterial pressure is transmitted to the venous system [59]. There are three types of this condition: type I, spinal dural arteriovenous fistula; type II, intramedullary arteriovenous malformation; type III, extradural–intradural arteriovenous malformations; and type IV, intradural perimedullary arteriovenous fistula.

## 3. Current Treatment Strategies and Their Limitations

Following the confirmation of a positive diagnosis, the formulation of a therapeutic strategy is entrusted to a multidisciplinary tumor board, comprising experts from various specialized departments including oncology, neurosurgery, radiation oncology, medical oncology, neurology, radiology, pathology, rehabilitation medicine, palliative care, and genetics [21,60]. A diverse array of treatments exists for spinal tumors, ranging from radiation therapy to extensive en bloc resection [13]. However, inherent limitations constrain their broad application and efficacy. In response, contemporary technological advancements, including nanotechnology, 3D printing, gene therapy, immunotherapy, and targeted therapy, coupled with novel surgical adjuncts and digital tools, are being increasingly integrated into spinal tumor management to address the shortcomings associated with conventional treatment approaches.

### 3.1. Surgical Method

The primary treatment approach for spinal cord tumors is surgical excision, which should be incorporated into the diagnostic and therapeutic strategy outlined by the tumor board [21,61,62,63]. Surgery aims to achieve maximal resection while minimizing the risk of long-term neurological dysfunction.

Precise access to intramedullary tumors is determined through careful evaluation of diagnostic imaging prior to surgery. Recent advancements in mixed reality (MR) technology, utilizing head-mounted displays (HMDs), enable surgeons to visualize stereoscopic images in 3D [64,65]. MR systems have demonstrated effectiveness in tumor removal by providing surgeons with enhanced visualization of vital structures, such as hidden blood vessels within the surgical field [66,67]. Moreover, utilizing HMDs to view spinal cord tumors in 3D enables surgeons to intuitively comprehend the tumor’s location, facilitating the preoperative planning of decompression methods and laminectomy sites [68].

Intraoperative ultrasonography (IOUS) is employed during surgeries for spinal intradural mass lesions to limit the extent of exposure, affecting not just dural incision and myelotomy but also bony exposure [69]. IOUS is capable of evaluating all types of mass lesions and can predict surgical outcomes. It enables the visualization of nerve rootlets and dentate ligaments, aiding in the mobilization of neural structures, particularly in cases with primarily anterior lesions. In situations involving highly infiltrative lesions where the tumor appears isoechoic to the spinal cord, making the tumor–myelin interface indistinct, IOUS proves invaluable in guiding surgical decisions [70]. However, the tool has its limitations, such as the size of the ultrasound probe tip (1 × 3.5 cm) sometimes exceeding the surgical field, and difficulty in visualizing parenchyma during heavy bleeding, as well as during the use of excessive hemostatic material, which appears highly hyperechoic [71].

The reliability of matching pathologic findings from frozen biopsies of spinal cord tumors with the final diagnosis remains controversial [72]. Consequently, reliance solely on frozen section results for surgical planning is inadequate; a comprehensive approach incorporating multiple factors—clinical characteristics, preoperative imaging, frozen-section diagnosis, and intraoperative tumor planes—is essential. Despite advancements in software [73], intraoperative ultrasound [74,75], and corrections made by intraoperative MRI [76], current technologies still fail to deliver the needed accuracy for consistent, precise, and extensive tumor resection [77,78]. New technologies are emerging that enable the accurate visualization of brain tumors and even residual tumor cells using fluorescent markers.

5-aminolevulinic acid (5-ALA) is a prodrug that accumulates in tumor cells, which convert it into protoporphyrin IX, a fluorescent compound [79,80]. This fluorescence allows surgeons to better visualize and distinguish tumor tissue from normal spinal cord tissue during surgery [81]. The benefits of using 5-ALA in spinal cord tumor surgery include enhanced visualization, which aids in maximal tumor removal, reduced risk of neurological damage, and the potential for better patient outcomes such as lower recurrence rates and improved quality of life. However, challenges include the possibility that not all tumor cells may fluoresce, variable sensitivity and specificity of the fluorescence, and the need for specialized training and experience in surgeons [82]. Current studies focus on improving the sensitivity and specificity of fluorescence, exploring its utility in different types of spinal tumors, and integrating it with other surgical technologies like intraoperative MRI and robotics.

The choice of surgical approach is straightforward, aiming for the shortest route to the tumor, given the absence of non-eloquent neural tissues within the spinal cord parenchyma. The three primary surgical accesses include the posterior median sulcus approach, the posterolateral sulcus approach, and the lateral direct subpial approach [83]. A posterior median sulcus approach is commonly used for most gliomas, including ependymomas and astrocytomas, while a lateral myelotomy is more appropriate for vascular tumors like hemangioblastomas or cavernous malformations, where the lesion is visible under microscopic examination [84,85,86].

Tumor staging is very important to treat patients with spinal vertebral tumors/metastases. However, the prognosis of patients with spinal tumors/metastases is not very promising and difficult to predict. Several systems are based on the overall tumor load and functional status of the patient and the anatomical extent of tumor involvement (Table 8, Table 9 and Table 10) [87,88,89]. Among them, Tomita and modified Tokuhashi prognosis scores have become very popular and are used to determine the optimal patient treatment. Tokuhashi et al. described a scoring system based on six parameters, which they later revised to take into account the stronger influence of the primary tumor type on survival [87]. The Tokuhashi score is better for predicting the short-term survival rate (Table 8). The primary tumor type is given more weight in the scoring system of Tomita et al. (Table 9) [88].

During surgery, the exposure is widened to fully reveal the intraparenchymal lesion, and a careful dissection plane is established between the tumor and the healthy spinal cord tissue. Typically, the tumor’s caudal and rostral boundaries are identified by cystic formations. Ependymomas manifest as encapsulated, brownish-red, sausage-shaped tumors, whereas astrocytomas appear as vague, whitish swellings accompanied by cysts.

Ependymomas display a red or dark gray coloration with distinct margins from the surrounding spinal cord tissue, allowing for precise separation along the craniocaudal axis before complete excision. These tumors commonly adhere ventrally to small vessels passing through the anterior median raphe, necessitating careful preservation of the anterior spinal artery [61,83,90,91]. Unlike ependymomas, astrocytomas typically lack a clear boundary between the tumor and the spinal cord. Removal of these tumors often involves piecemeal excision, beginning with internal decompression and progressing outward to define the tumor–cord interface [92]. Hemangioblastomas, which are subpial and highly vascular, contain small arterial feeders that end within the tumor. These are excised en bloc by coagulating the feeder vessels near the tumor’s surface [93].

Once the interface between the tumor and the spinal cord is established, the tumor can be carefully debulked using gentle dissection or ultrasonic cavitation to reduce manipulation of the spinal cord. Nonetheless, achieving a complete resection of benign tumors without causing neurological damage is challenging, especially when the tumor–spinal cord interface is not clearly defined [92].

Intraoperative neuromonitoring plays a vital role in surgical resections. Its outstanding predictive capabilities regarding functional outcomes have established it as an essential tool in all spinal cord tumor surgeries. Mehta et al. demonstrated that dorsal column dysfunction occurred in just 9% of patients monitored with SSEP, compared with 50% in those without SSEP monitoring [94].

### 3.2. Radiotherapy

Radiotherapy is typically reserved for situations where en bloc resection is unfeasible. Traditionally, high doses of radiation (40–60 Gy) are required, leading to a high incidence of complications due to the proximity of the spinal cord and thoracoabdominal organs, including radiation myelopathy and various issues affecting gastrointestinal and reproductive health, e.g., hormonal imbalances, reduced fertility, uterine dysfunction, miscarriage, preterm labor, low birth weight, and placental abnormalities [39,43,95]. However, with the advent of intensity-modulated radiation therapy and stereotactic radiosurgery, it is now possible to deliver high radiation doses directly to the spinal region while sharply reducing exposure to surrounding areas, thereby minimizing the side effects typical of conventional radiation treatments [96,97].

According to a study by Shin et al., stereotactic radiosurgery (SRS) proves to be an effective treatment for benign neurogenic tumors, though malignant spinal neurogenic tumors (MPNSTs) exhibit variable responses to SRS [98]. Other case series have similarly reported excellent rates of local control with minimal neurotoxicity [99,100]. Thus, while surgical removal continues to be the preferred treatment for most intradural tumors, radiosurgery emerges as a viable alternative, particularly for recurrent, residual, or multiple lesions (such as in familial phakomatoses), or when surgical intervention is contraindicated or ill-advised due to patient comorbidities or poor health [100].

### 3.3. Systemic Therapy

Chemotherapy is less effective in treating spinal cord astrocytomas than intracranial ones [101,102]. Several studies have reported only a partial response to temozolomide in spinal cord astrocytomas [103,104,105]. Furthermore, pediatric low-grade astrocytoma patients with adjuvant radiation therapy and chemotherapy after subtotal resection had longer survival than those who had only a subtotal resection or en bloc resection [106]. Some reports suggest that etoposide might benefit patients with recurrent spinal cord ependymomas [107]. Targeted therapies for ependymomas are under investigation. Imatinib indicated the potential of recurrent spinal cord ependymomas with overexpressed platelet-derived growth factor (PDGF), though such overexpression is not present [108]. Bevacizumab has been showed to reduce the size of cystic spinal cord ependymomas in NF2 patients, with clinical improvement observed [109].

Medical therapy trials for NF2-related schwannomas have had limited success, particularly with agents targeting epidermal growth factor receptor (EGFR) activity. Lapatinib, a selective EGFR inhibitor, showed some efficacy in ependymomas, although follow-up outcomes have been inconsistent [110,111,112]. However, despite their potential to curb tumor growth, chemotherapeutic agents must be administered at high systemic doses to achieve effective concentrations at tumor sites, adversely affecting normal tissue health and leading to side effects such as neurotoxicity, kidney toxicity, and cardiac toxicity.

## 4. Emerging Treatment Strategies

### 4.1. Immunotherapy 

Immunotherapeutic strategies focus on activating the patient’s immune system to target and destroy cancer cells, preventing them from evading or reaching a balance with the immune system [20,39]. Current leading immunotherapy treatments for gliomas include checkpoint inhibitors, cancer vaccines, and chimeric antigen receptor T cells (CAR-T cells). Notably, CAR-T cell therapy involves modifying allogeneic or autologous T cells in vitro to carry CAR molecules on their membranes [113]. These modified T cells are reintroduced into the patient’s body to target and destroy tumor cells that express the specific antigen. There is increasing interest in applying immunotherapy to several gliomas, with developments in CAR-T therapy, immune checkpoint inhibitors, and vaccine-based strategies. Because of the rarity of spinal cord gliomas, obstacles in crossing the blood–spinal cord barrier, limited antigens for targeting, and potential neurotoxic side effects impede the use of immunotherapy in these tumors [20]. Furthermore, immune checkpoint inhibitors can lead to autoimmune diseases and, in severe cases, death [114]. The selection of spinal cord tumor patients for treatment with immunotherapy or immune checkpoint inhibitors remains a challenge. Nowadays, several approaches have been reported [18,115,116]. For high-grade spinal cord astrocytomas, the prognosis is often poor with the currently available therapies. Immunotherapy is at the experimental stage in such gliomas. However, recent novel advances in immunotherapy include immune checkpoint inhibitors, chimeric antigen receptor (CAR)-T therapy, and vaccine therapy [115]. Immunotherapies targeting the programmed cell death-1 receptor (PD-1) and its ligand-1 (PD-L1) yielded impressive clinical results in advanced malignant tumors expressing high levels of PD-L1 [116]. Immunotherapy has the potential to play an increasingly important role in the treatment of these tumors. Several clinical trials have evaluated immunotherapy for intracranial gliomas, providing evidence for an immunotherapy-mediated ability to inhibit tumor growth [117].

### 4.2. Neural Stem Cells

Neural stem cells (NSCs) are pluripotent cells with the capability to develop into either gliogenic or neurogenic lineages [118]. A notable characteristic of NSCs is their inherent tropism for tumors in vivo, which positions them as excellent vehicles for targeted cancer therapies [119,120]. One innovative approach involves engineering NSCs to produce an enzyme that activates a harmless prodrug, which then transforms into a potent chemotherapeutic agent near the tumor cells [121]. This strategy has shown potential, particularly in initial studies where NSCs were modified to express cytosine deaminase. This enzyme converts the prodrug 5-FC into the active chemotherapeutic, 5-fluorouracil, effectively targeting and reducing tumor size in glioblastoma models in rodents through the bystander effect [119]. Further research includes a dual-gene strategy where NSCs are engineered to express both cytosine deaminase and thymidine kinase, the latter converting the prodrug ganciclovir into the oncolytic agent ganciclovir triphosphate, enhancing the therapeutic impact [122].

### 4.3. Cancer Vaccine

Cancer vaccines are formulated from antigens that are mainly expressed on specific cancer cells [123]. These antigens provoke an immune response aimed at selectively eliminating the targeted cell. Nowadays, there are five antitumor vaccine therapies, with peptide vaccines and dendritic cell (DC) vaccines being the two basic approaches [124]. Peptide vaccines for spinal gliomas are composed of 8–30 amino acids or tumor-specific antigens such as isocitrate dehydrogenase (IDH)-1(R132H) and EGFRvIII [123,125]. Conversely, autologous dendritic cell vaccines are developed ex vivo by culturing CD14+ monocytes with IL-4 and granulocyte-macrophage colony-stimulating factor (GM-CSF) [125]. These vaccines are primed with tumor-specific antigens. Several phase I and II clinical trials have documented the effectiveness and safety of DC vaccines to treat malignant gliomas [126,127,128,129].

### 4.4. Tumor-Targeted Therapies (Nanotechnology)

Investigations into the local environment of neoplastic diseases have led to the discovery and detailed study of the tumor microenvironment (TME), enhancing our understanding of cancer progression and fostering the development of more targeted therapies. Recent advancements include the development of several nanoformulations aimed at delivering targeted therapies specifically for spinal cord tumors and spinal metastases. A notable study by Yan et al. [130] involved a novel bone-targeted protein nanomedicine that combines saporin with a boronated polymer, encapsulated in an anionic poly(aspartic acid) layer. In mouse models, these nanoparticles accumulated in the bone and released saporin in response to the acidic tumor environment, effectively inactivating ribosomes and inducing cancer cell death.

For intramedullary spinal cord tumors (IMSCTs), magnetic nanoparticles loaded with doxorubicin have been developed by Kheirkhah et al. [131], showing targeted delivery and localized chemotherapeutic-induced apoptosis in cancer cells. Ahmadi et al. [132] have explored an advanced anticancer formulation using methotrexate encapsulated in a smart nanocarrier featuring a magnetic core and a polymeric shell with cationic properties. Huang et al. [133] have engineered nanoparticles that merge the benefits of exosomes with lncRNA MEG3 to target four human osteosarcoma cell lines, including MNNG/HOS, U2OS, MG63, and SaOS-2, showcasing another innovative approach to cancer treatment.

## 5. Conclusions

Spinal cord tumors are a rare and complex group of neoplasms that can be challenging to diagnose and treat. This review article discussed the current state of knowledge on spinal cord tumors, including their classification, diagnosis, treatment, and emerging therapeutic strategies. This article highlighted the importance of a multidisciplinary approach to care, which involves neurosurgeons, radiation oncologists, medical oncologists, neurologists, radiologists, pathologists, rehabilitation specialists, and palliative care physicians. Current treatment strategies for spinal cord tumors include surgery, radiation therapy, and chemotherapy. However, these treatments can be limited by their side effects and the difficulty of targeting tumors in the spinal cord. Emerging therapeutic strategies, such as immunotherapy, neural stem cell therapy, cancer vaccines, and tumor-targeted therapies, offer promise for improving the outcomes of patients with spinal cord tumors.

## Figures and Tables

**Table 3:** Differential diagnosis tips.

Tumor location
MRI intensity and CT density
Enhancement pattern
Bone erosion
Accompanied findings (peritumoral cyst, edema, flow void, calcification, etc.)
Other studies (angiography, PET, CSF study, etc.)

**Table 4:** The key points of differential diagnosis of meningioma and schwannoma.

Category	Meningioma	Schwannoma
T2-weighted MR imaging	Iso-low	High; heterogenous
Enhanced	Homogenous	Heterogenous
Location	Lateral	Posterior
Cyst	−~+	++
Calcification	−~+	−
Tumor angle	Dull	Sharp
Dural tail	+	−
Mobile tumor	−	+

**Table 7:** Wilkins and Odom’s neurenteric cyst histopathological classification system [51].

Characteristics	Type A	Type B	Type C
Single layer of pseudostratified columnar or cuboidal cells mimicking respiratory or gastrointestinal epithelium	+	+	+
Complex invaginations with glandular organization; mucinous or serous production; and nerve ganglion, lymphoid, skeletal muscle, smooth muscle, fat, cartilage, and/or bone elements	−	+	−
Ependymal or glial tissue	−	−	+

**Table 8:** Tokuhashi score [87].

Prognosis Parameter	Score
Patient condition Poor (performance status: 10–40%) Moderate (performance status: 50–70%) Good (performance status: 80–100%)	0 1 2
No. of bone metastases outside spine Poor (performance status: 10–40% Moderate (performance status: 50–70%) Good (performance status: 80–100%)	0 1 2
No. of bone metastases outside spine >2 1–2 0	0 1 2
Metastasis to major organs Nonremovable Removable None	0 1 2
Primary site Lung; osteosarcoma; stomach; bladder; esophagus; pancreas Liver; gallbladder; unidentified Other Kidney; uterus Rectum Thyroid; breast; prostate; carcinoid tumor	0 1 2 3 4 5
Palsy Complete (Frankel A; B) Incomplete (Frankel C; D) None (Frankel E)	0 1 2

**Table 9:** Tomita surgical classification for spinal malignant tumors [88].

Intra-compartmental	Type 1	Vertebral body
	Type 2	Pedicle extension
	Type 3	Body-lamina extension
Extra-compartmental	Type 4	Epidural extension
	Type 5	Paravertebral extension
	Type 6	2-3 vertebrae
Multiple	Type 7	Multiple, more than 2

**Table 10:** Enneking staging for malignant musculoskeletal tumors [89].

Stage	Grade	Site	Metastasis
IA	Low (G1)	Intra-compartmental (T1)	No metastasis (M0)
IB	Low (G1)	Extra-compartmental (T2)	No metastasis (M0)
IIA	High (G2)	Intra-compartmental (T1)	No metastasis (M0)
IIB	High (G2)	Extra-compartmental (T2)	No metastasis (M0)
III	Any (G)	Any (T)	Regional or distant metastasis (M1)
